# Supplementary figures and images for: Scanning Electron Microscopy Reveals New Ultrastructural Features in Metacercariae of Clinostomum cutaneum (Digenea: Clinostomidae) Infecting Oreochromis niloticus (Actinopterygii: Cichlidae) in Kenya
Source: Pathogens. 2025 Mar 4;14(3):249. doi: 10.3390/pathogens14030249 (PMC11944436; doi:10.3390/pathogens14030249)

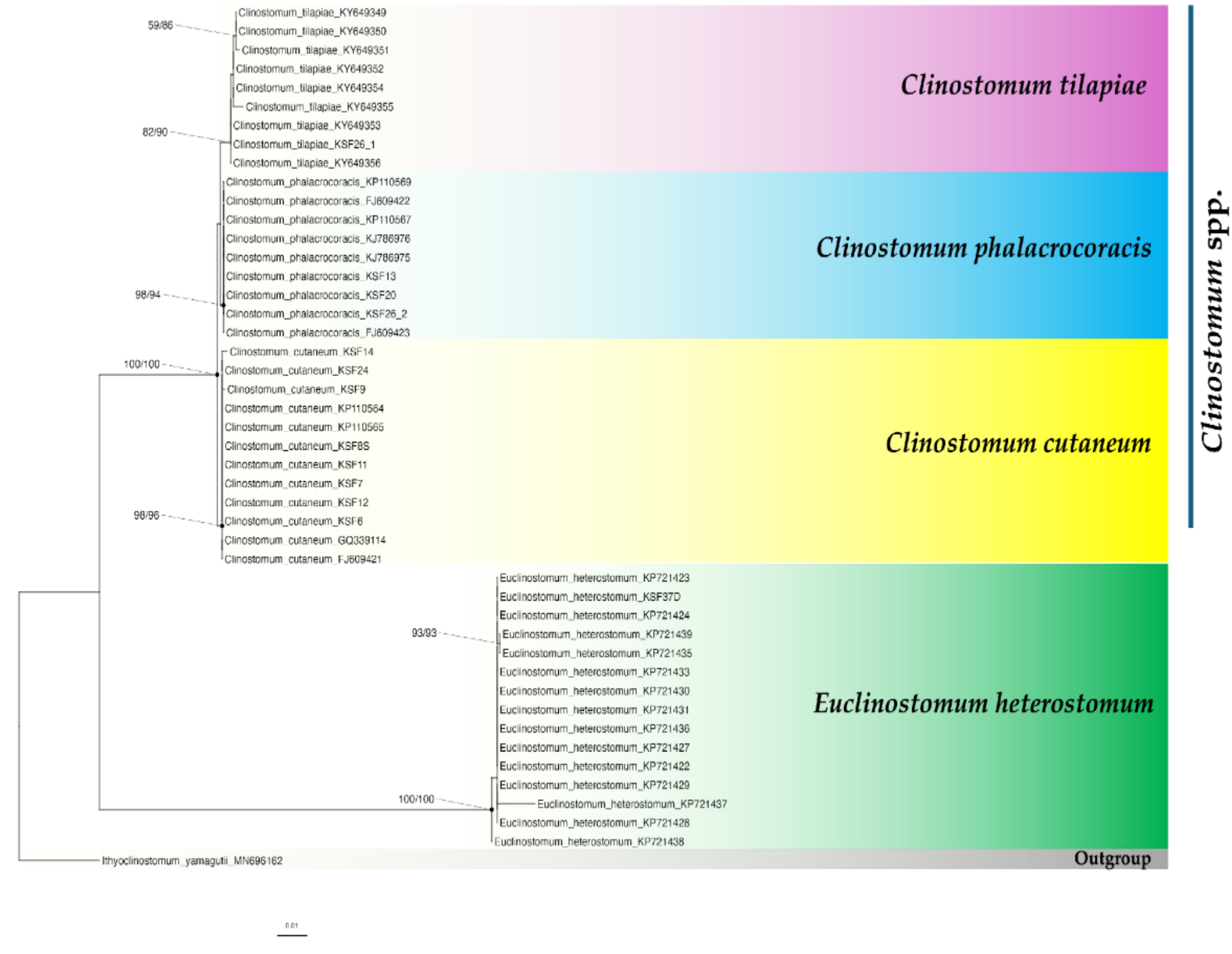

Supplement: Supplementary file 1 [file pathogens-14-00249-s001.zip › pathogens-3485539-supplementary.png]
